# Complete genome sequence of *Jiangella gansuensis* strain YIM 002^T^ (DSM 44835^T^), the type species of the genus *Jiangella* and source of new antibiotic compounds

**DOI:** 10.1186/s40793-017-0226-6

**Published:** 2017-02-03

**Authors:** Jian-Yu Jiao, Lorena Carro, Lan Liu, Xiao-Yang Gao, Xiao-Tong Zhang, Wael N. Hozzein, Alla Lapidus, Marcel Huntemann, T. B. K. Reddy, Neha Varghese, Michalis Hadjithomas, Natalia N. Ivanova, Markus Göker, Manoj Pillay, Jonathan A. Eisen, Tanja Woyke, Hans-Peter Klenk, Nikos C. Kyrpides, Wen-Jun Li

**Affiliations:** 1 State Key Laboratory of Biocontrol and Guangdong Provincial Key Laboratory of Plant Resources, College of Life Science, Sun Yat-Sen University, Guangzhou, China; 2 School of Biology, Newcastle University, Newcastle upon Tyne, UK; 3 Key Laboratory of Tropical Plant Resources and Sustainable Use, Xishuangbanna Tropical Botanical Garden, Chinese Academy of Science, Yunnan Province, China; 4 Bioproducts Research Chair (BRC), College of Science, King Saud University, Riyadh, Kingdom of Saudi Arabia; 5 Lawrence Berkeley National Laboratory, Berkeley, CA 94720 USA; 6 Center for Algorithmic Biotechnology, St. Petersburg State University, St. Petersburg, Russia; 7 DOE Joint Genome Institute, Walnut Creek, CA USA; 8 Leibniz-Institute DSMZ – German Collection of Microorganisms and Cell Cultures, Braunschweig, Germany; 9 Biological Data Management and Technology Center, Lawrence Berkeley National Laboratory, Berkeley, CA USA; 10 University of California, Davis, CA USA; 11 Department of Biological Sciences, Faculty of Science, King Abdulaziz University, Jeddah, Saudi Arabia; 12 Botany and Microbiology Department, Faculty of Science, Beni-Suef University, Beni-Suef, Egypt; 13 Key Laboratory of Biogeography and Bioresource in Arid Land, Xinjiang Institute of Ecology and Geography, Chinese Academy of Sciences, Urumqi, China

**Keywords:** *Jiangella gansuensis*, *Jiangellales*, Desert, Genome, Taxonomic comments, GEBA

## Abstract

*Jiangella gansuensis* strain YIM 002^T^ is the type strain of the type species of the genus *Jiangella*, which is at the present time composed of five species, and was isolated from desert soil sample in Gansu Province (China). The five strains of this genus are clustered in a monophyletic group when closer actinobacterial genera are used to infer a 16S rRNA gene sequence phylogeny. The study of this genome is part of the ***G*** *enomic* ***E*** *ncyclopedia of* ***B*** *acteria and* ***A*** *rchaea* project, and here we describe the complete genome sequence and annotation of this taxon. The genome of *J. gansuensis* strain YIM 002^T^ contains a single scaffold of size 5,585,780 bp, which involves 149 pseudogenes, 4905 protein-coding genes and 50 RNA genes, including 2520 hypothetical proteins and 4 rRNA genes. From the investigation of genome sizes of *Jiangella* species, *J. gansuensis* shows a smaller size, which indicates this strain might have discarded too much genetic information to adapt to desert environment. Seven new compounds from this bacterium have recently been described; however, its potential should be higher, as secondary metabolite gene cluster analysis predicted 60 gene clusters, including the potential to produce the pristinamycin.

**Electronic supplementary material:**

The online version of this article (doi:10.1186/s40793-017-0226-6) contains supplementary material, which is available to authorized users.

## Introduction

*Jiangella gansuensis* strain YIM 002 ^T^ (=DSM 44835 ^T^ =CCTCC AA 204001 ^T^ =KCTC 19044 ^T^) is the type strain of *J. gansuensis*. This organism is an aerobic, Gram-positive, haloduric filamentous actinomycete, placed within the genus *Jiangella* [1].

The genus *Jiangella* was first identified by Song et al. in 2005, including five halotolerant species listed at present by LPSN [2]. Members of this taxon isolated from different habitats, respectively, are rarely described except for their polyphasic approach based on combination of phenotypic and genotypic characteristics [1, 3–6]. The *Jiangella* was originally identified as a new genus of the family *Nocardioidaceae* within the suborder *Propionibacterineae* [1] based on phenotypic and genotypic criteria. However, the reconstruction of the phylogenetic relationships of *Actinobacteria* at higher taxa was done later based on using the 16S rRNA genes and other related evidences, such as taxon-specific 16S rRNA gene signature nucleotides [7, 8]. After the genus *Haloactinopolyspora* was described by Tang et al., the genus *Jiangella* together with the genus *Haloactinopolyspora* were placed in a novel family *Jiangellaceae* belong to *Jiangellineae* subord. nov., mainly because of theirs signature nucleotide patterns, 16S rRNA gene similarity and phylogenetic criteria [9]. Presently, the *J. gansuensis* is placed in the family *Jiangellaceae* of the order *Jiangellales* within the class *Actinobacteria* [10].

The capacity of *J. gansuensis* YIM 002 ^T^ to produce seven new compounds (five pyrrol-2-aldehyde compounds, jiangrines A-E; one indolizine derivative, jiangrine F; one glycolipid, jiangolide) has previously been shown [11], highlighting the importance of this bacterium and its analysis as a novel source of secondary metabolites. As part of the GEBA project and considering its phylogenetic position and biological significance, we finally decided to sequence the genome of the type strain of *J. gansuensis*. Here we present a summary classification and a set of features for *J. gansuensis* YIM 002 ^T^, together with the description of genomic sequencing and annotation. At the same time, we will provide a brief introduction of its genome in this article.

## Organism information

### Classification and features

**Fig. 1:**
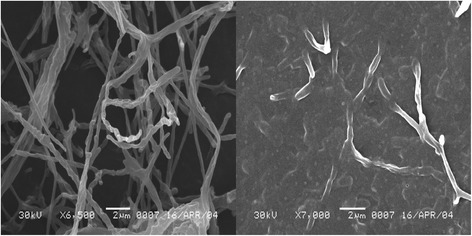
Scanning electron micrograph of *Jiangella gansuensis* strain YIM 002^T^ grown on ISP medium 2 for 14d at 28 °C. Bar size: 2 μm

Strain YIM 002 ^T^ is a free-living isolate collected from a desert soil sample of Gansu Province during an investigation into microbial diversity of extreme environments. This actinobacterium forms well-differentiated non-sporulating aerial and substrate mycelia. Its aerial hypha was observed to have yellow-white color at the earliest and finally turns to orange-yellow after few days on NA medium, and its substrate mycelia fragmented into short or elongated rods in the early phase of the growth (Fig. 1). Growth was observed on ISP 2, ISP 3, ISP 4, ISP 5, nutrient agar and Czapek’s agar [1, 12]. The type strain of this taxon is able to tolerate a pH range between 5.0 and 10.0, and able to growth at the salinity between 0 and 10% (*w/v* NaCl), with no growth observed at 12.5%. Optimal growth of strain YIM 002 ^T^ occurs at pH 7.0–8.0, 1–5% (*w/v*) NaCl and 28 °C. The diamino acid in the peptidoglycan is LL-2,6-diaminopimelate. MK-9(H_4_) is the predominant menaquinone. The primary phospholipids profile of strain DSM 44835 ^T^ was found to consist of phosphatidylinositol mannosides, phosphatidylinositol and diphosphatidylglycerol. Its major cellular fatty acids (>10%) are anteiso-C_15:0_, anteiso-C_17:0_ and iso-C_15:0_. Whole cell sugar composition includes glucose and ribose, whereas the amino acids in the peptidoglycan layer were LL-A_2_pm, alanine, glycine and glutamic acid [1]. The DNA G + C content of the type strain was previously determined as 70% while genome analysis showed a higher value of 70.91%.

The draft genome of *J. gansuensis* YIM 002 ^T^ has one almost full-length 16S rRNA gene sequence, which correspond perfectly with the original sequence from the species description (AY631071). The comparison of this 16S rRNA sequence of YIM 002 ^T^ using the EzTaxon-e server [13], showed highest similarity to *Jiangella alba* YIM 61503 ^T^ (98.93%), with close relationships to other species within the genus, *Jiangella muralis* 15-Je-017^T^ (98.88%), *Jiangella mangrovi* 3SM4-07^T^ (98.49%) and *Jiangella alkaliphila* D8-87^T^ (98.10%). Closest other genera are *Haloactinopolyspora* [9] and *Phytoactinopolyspora* [14]. The strains of the genus *Jiangella* have many 16S rRNA gene signature nucleotides compared with most of other described actinomycetes. This allows for distinguished them easily from other actinobacteria, especially in 11 unique positions, including 127:234 (G-C), 598:640 (C-G), 672:734 (G–C), 831:855 (U–A), 833:853 (G–C), 840:846 (A–U), 950:1231 (G–C), 952:1229 (G–C), 955:1225 (G–U), 986:1219 (U–G) and 987:1218 (C–G) [9].

**Fig. 2:**
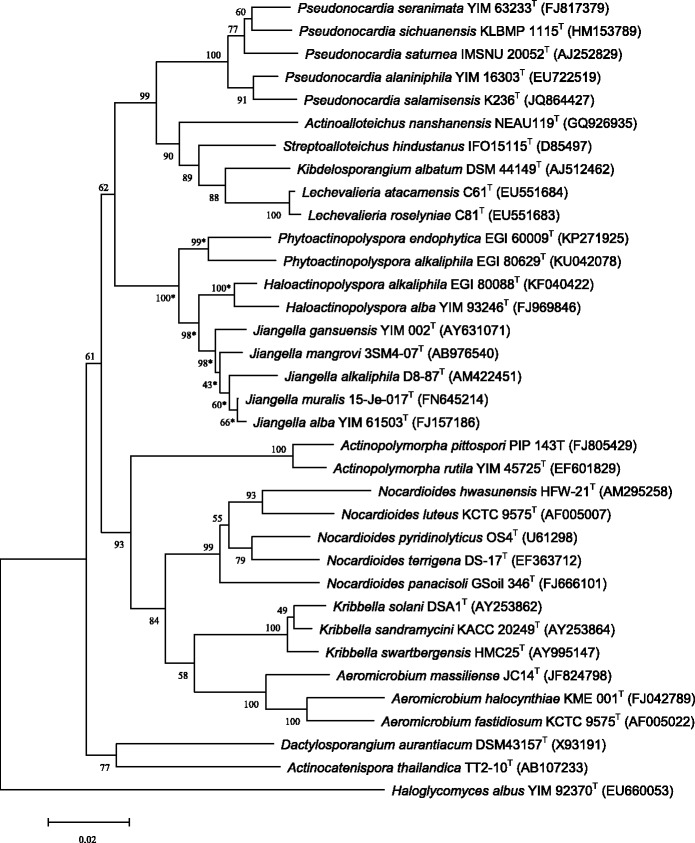
Phylogenetic tree showing the relationship of *J. gansuensis* YIM 002^T^ with some other actinobacteria based on 16S rRNA gene sequences. The Neighbour-joining tree was built using MEGA 5 [39] with the Kimura 2-parameter model. Bootstrap values (percentages of 1000 replicates) are shown at branch points. *Asterisks* denote nodes that were also recovered using the Maximum Likelihood method in the branch of *Jiangellaceae*. The *Haloglycomyces albus* act as the outgroup

Phylogenetic analyses were performed using both neighbor-joining (NJ) and maximum-likehood (ML) algorithms. The NJ phylogenetic tree of the genus *Jiangella* based on 16S rRNA genes provide an evidence of its independent taxon (Figs. 2 and Additional file 1: Figure S1), together with the genera *Haloactinopolyspora* and *Phytoactinopolyspora*, which arouse ours reflection on the relationship of three families among *Jiangellaceae*, *Nocardioidaceae* and *Pseudonocardiaceae*. The ML tree (Additional file 1: Figure S1) demonstrates the same positions in *Jiangellaceae* compared with the NJ tree. Minimum Information about the Genome Sequence is provided in Table 1.

**Table 1:** Classification and general features of *Jiangella gansuensis* strain YIM 002^T^ in accordance with the MIGS recommendations [20], List of Prokaryotic names with Standing in Nomenclature [40] and the Names for Life database [41]

MIGS ID	Property	Term	Evidence code^a^
	Current classification	Domain *Bacteria*	TAS [42]
		Phylum *Actinobacteria*	TAS [43]
		Class *Actinobacteria*	TAS [7]
		Order *Jiangellales*	TAS [44]
		Family *Jiangellaceae*	TAS [9]
		Genus *Jiangella*	TAS [1]
		Species *Jiangella gansuensis*	TAS [1]
		Type strain YIM 002^T^ (=DSM 44835^T^)	TAS [1]
	Gram stain	Positive	IDA
	Cell shape	Filamentous	IDA
	Motility	Non motile	IDA
	Sporulation	Non-sporulating	IDA
	Temperature range	10–45 °C	IDA
	Optimum temperature	28 °C	IDA
	pH range; Optimum	7.0–8.0	TAS [1]
	Carbon source	Various	IDA
	Energy source	Chemoorganotroph	IDA
MIGS-6	Habitat	Desert soil	IDA
MIGS-6.3	Salinity	Halotolerant	IDA
MIGS-22	Oxygen requirement	Aerobic	IDA
MIGS-15	Biotic relationship	Free living	IDA
MIGS-14	Pathogenicity	None	IDA
MIGS-4	Geographic location	Gansu Province, China	IDA
MIGS-5	Sample collection time	2005 or before	NAS
MIGS-4.1	Latitude	Not reported	NAS
MIGS-4.2	Longitude	Not reported	
MIGS-4.4	Altitude	Not reported	

^a^ Evidence codes - IDA: Inferred from Direct Assay; TAS: Traceable Author Statement (i.e., a direct report exists in the literature); NAS: Non-traceable Author Statement (i.e., not directly observed for the living, isolated sample, but based on a generally accepted property for the species, or anecdotal evidence). These evidence codes are from of the Gene Ontology project [45]

## Genome sequencing information

### Genome project history

This organism was selected for sequencing on the basis of its important phylogenetic position and biological significance [15, 16], and for a better understanding of the school of ‘evolutionary taxonomy’ [17]. Sequencing of *J. gansuensis* YIM 002 ^T^ is part of Genomic Encyclopedia of Bacteria and Archaea pilot project [18], which aims for generating high quality draft genomes for bacterial and archaeal strains. The genome project is deposited in the Genomes OnLine Database (GOLD) [19], and the finished genome sequence was deposited in GenBank. Genome sequencing, finishing and annotation were performed by the Department of Energy, Joint Genome Institute (JGI) using state of the art genome sequencing technology [20]. A summary of project information is shown in Table 2, compliance with MIGS version 2.0 [21].

**Table 2:** Genome sequencing project information

MIGS ID	Property	Term
MIGS 31	Finishing quality	Non-contiguous Finished
MIGS-28	Libraries used	Illumina Std shotgun library
MIGS 29	Sequencing platforms	454-GS-FLX-Titanium Illumina GAii
MIGS 31.2	Fold coverage	Unknown
MIGS 30	Assemblers	ALLPATHS v. R37654
MIGS 32	Gene calling method	Prodigal 1.4, GenePRIMP
	Locus Tag	JIAGA
	GenBank ID	AZXT00000000
	GenBank Date of Release	15-08-2013
	GOLD ID	Gp0001209
	BIOPROJECT	PRJNA224116, PRJNA63165
MIGS 13	Source Material Identifier	YIM 002, DSM 44835
	Project relevance	Tree of Life, GEBA

### Growth conditions and genomic DNA preparation

*J. gansuensis* strain YIM 002 ^T^ (=DSM 44835 ^T^) was grown in DSMZ medium 65 (GYM *Streptomyces* medium) at 28 °C. Genomic DNA was isolated using Qiagen Genomic 500 DNA Kit (Qiagen, Hilden, Germany) following the standard protocol provided by the manufacturer. Some modifications were included for cell lysis, first freezing for 20 min (−70 °C), then heating 5 min (98 °C), and cooling 15 min to 37 °C; adding 1.5 ml lysozyme (standard: 0.3 ml, only), 1.0 ml achromopeptidase, 0.12 ml lysostaphine, 0.12 ml mutanolysine, 1.5 ml proteinase K (standard: 0.5 ml, only), followed by overnight incubation at 35 °C.

### Genome sequencing and assembly

**Fig. 3:**
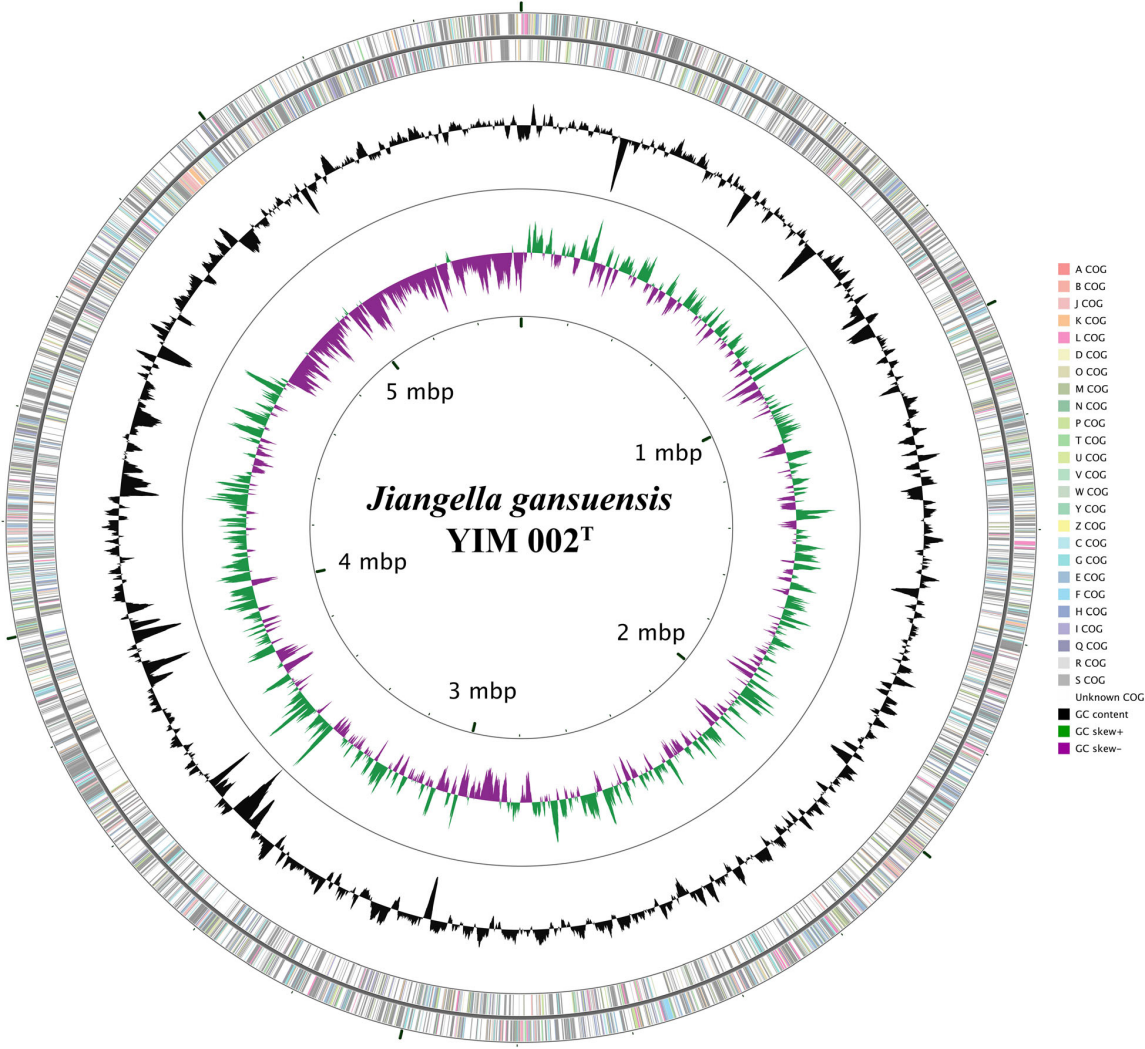
Graphical map of the *J. gansuensis* strain YIM 002^T^ chromosome. The genome circular map was set up by the CGView Server [46]. From the outside to the center: Genes on forward strand (colored by COG categories), Genes on reverse strand (colored by COG categories), GC content, GC skew, where *green* indicates positive values and *magenta* indicates negative values

All general aspect of library construction and sequencing performed can be found at the JGI website. The complete sequence in one scaffold was obtained from 9 contigs with the assembly method ALLPATHS v. R37654, obtaining a total size of 5.5 Mbp from a total volume data of 4 Gbases (Fig. 3).

### Genome annotation

Prodigal [22] was used to identify genes as part of the JGI genome annotation pipeline [23, 24] followed by a round of manual curation using the JGI GenePRIMP pipeline [25]. The National Center for Biotechnology Information non-redundant database, UniProt, TIGR/Fam, Pfam, PRIAM, KEGG, COG, and InterPro databases were used to analyse the predicted CDSs after translation. RNA genes identification was done using HMMER 3.0 [26] (rRNAs) and tRNAscan-SE 21.23 [27] (tRNAs). INFERNAL 1.0.2 [28] was used for prediction of other non-coding genes. Integrated Microbial Genomes Expert Review platform [29] permitted the additional gene prediction analysis and functional annotation. CRISPR elements were detected with CRT [30] and PILER-CR [31]. General statistics are shown in Table 3.

**Table 3:** Genome Statistics

Attribute	Value	% of total ^a^
Genome size (bp)	5,585,780	100.00
DNA coding (bp)	4,761,339	85.24
DNA G + C (bp)	3,960,974	70.91
DNA scaffolds	1	-
Total genes	5,104	-
Protein-coding genes	4,905	98.03
RNA genes	50	0.98
Pseudo genes	149	2.98
Genes in internal clusters	1763	34.54
Genes with function prediction	2,504	48.86
Genes assigned to COGs	2,156	42.07
Genes with Pfam domains	1,734	33.97
Genes with signal peptides	456	8.69
Genes with trandmembrane helices	1230	23.43
CPISPR repeats	0	-

^a^ The total is based on either the size of genome in base pairs or the total number of genes in the predicted genome

## Genome properties

The assembly of the draft genome sequence consists of one scaffold for the strain YIM 002 ^T^ (Fig. 1), with 70.9% GC content (Table 3) in 5,585,780 nucleotides. From a total of 5104 genes, there were 4905 protein-coding genes, 149 pseudogens and 50 RNA genes. Numbers of the genes were assigned a putative function (48.86%), while the remaining protein-coding genes were annotated as hypothetical proteins. COGs categories distributions for the genes are presented in Table 4.

**Table 4:** Number of genes associated with the general COG functional categories

Code	Value	% age ^a^	Description
J	160	3.18	Translation, ribosomal structure and biogenesis
A	1	0.02	RNA processing and modification
K	230	4.58	Transcription
L	116	2.31	Replication, recombination and repair
B	1	0.02	Chromatin structure and dynamics
D	21	0.42	Cell cycle control, cell division, chromosome partitioning
V	60	1.19	Defence mechanisms
T	75	1.49	Signal transduction mechanisms
M	96	1.91	Cell wall/membrane biogenesis
N	0	0.00	Cell motility
U	18	0.36	Intracellular trafficking, secretion, and vesicular transport
O	69	1.37	Posttranslational modification, protein turnover, chaperones
C	160	3.18	Energy production and conversion
G	223	4.44	Carbohydrate transport and metabolism
E	298	5.93	Amino acid transport and metabolism
F	56	1.11	Nucleotide transport and metabolism
H	114	2.27	Coenzyme transport and metabolism
I	111	2.21	Lipid transport and metabolism
P	179	3.56	Inorganic ion transport and metabolism
Q	84	1.67	Secondary metabolites biosynthesis, transport and catabolism
R	311	6.19	General function prediction only
S	151	3.00	Function unknown
-	2868	57.09	Not in COGs

^a^The total is based on the total number of protein-coding genes in the genome

## Insights from the genome sequence

The genome of YIM 002 ^T^ with a high G + C content and the smallest size within the *Jiangella* genomes (Table 3) may be the result of selection and mutation [32], which could involve several factors, such as environment, aerobiosis and others [33]. Generally speaking, a larger genome size may correlate with more complex habitat, suggesting that the genome encodes a large metabolic and stress-tolerance potential [34]. However, after we investigated the genome size of other type strains of *Jiangella* species, we found the size of the other three strains sequenced of this genus, *J. alkaliphila*, *J. alba* and *J. muralis* greater than 7 Mbp based on the genome data from NCBI. This result could implicate that the tight packing and small size of *J. gansuensis* is likely an adaptation for reproductive efficiency or competitiveness [35]. As a halotolerant actinobacterium, solute and ion transporter were predicted in its genome. At the same time, the genome shows properties related to solution of nitrate and sulfonate transport systems. Moreover, nitrite reductase and nitrogen fixation protein NifU were also detected.

The capacity of this microorganism to produce antibiotics has been recently proved with the description of seven new compounds (five pyrrol-2-aldehyde compounds, jiangrines A-E; one indolizine derivative, jiangrine F; one glycolipid, jiangolide) [11]. However, its potential should be higher, taken account the 45 biosynthetic clusters found within the JGI tool [36] and the 497 genes implicated in these clusters. As most of the clusters appear to be putative genes in this analysis, a second approach was carried out to detect the variety of biosynthetic types and enhance manual genome annotations of secondary metabolite biosynthesis. The software pipeline antiSMASH for secondary metabolite gene cluster identification, annotation and analysis was used [37, 38]. From this analysis, 60 gene clusters were identified, including 20 gene clusters in which the most similar clusters were still unknown (Additional file 2: Table S1). The result of the analysis shown the potential of *J. gansuensis* to produce pristinamycin, an antibiotic derived from *Streptomyces* *pristinaespiralis* effective against staphylococcal infections, and other antibiotics.

## Conclusions

The genome sequence and annotation of *J. gansuensis* YIM 002 ^T^ were presented. This draft genome possess a smaller size (5.59 Mb) compared with other *Jiangella* species, and contents 2504 function predicted proteins, indicating that *J. gansuensis* possibly discarded many genes to adapt to the extreme desert conditions during its evolution. Although the processes of nitrous metabolism and secondary metabolism need further investigation to fully understand the related pathways, we believe that *J. gansuensis* participates in nitrogen cycling and has an important ability to produce secondary metabolites. This genome will contribute to further studies on phylogenetics and the mechanisms of environmental adaptation. A combined study together with genomes of other members in the family *Jiangellaceae* will help us to better understand the ecological role of this taxon and its relationships to other actinobacteria.

## Additional files

Additional file 1: Figure S1. Phylogenetic tree showing the relationship of *J. gansuensis* YIM 002^T^ with some other actinobacteria based on 16S rRNA sequences. The maximum-likelihood tree was built using MEGA 5 [39]. Bootstrap values (percentages of 1000 replicates) are shown at branch points. *Haloglycomyces albus* was used as outgroup. (PDF 92 kb)
Additional file 2: Table S1. Number of gene clusters associated with antiSMASH. (DOCX 73 kb)

## Abbreviations

CRISPR: Clustered regularly interspaced short palindromic repeats
GEBA: Genomic encyclopedia of bacteria and archaea
IMG-ER: Integrated microbial genomes – expert review
JGI: Joint Genome Institute
LPSN: List of prokaryotic names with standing in nomenclature
ML: Maximum likelihood
NJ: Neighbour joining

## References

[1] Song L, Li WJ, Wang QL, Chen GZ, Zhang YS, Xu LH. *Jiangella gansuensis* gen. nov., sp. nov., a novel actinomycete from a desert soil in north-west China. Int J Syst Evol Microbiol. 2005;55:881–4. 10.1099/ijs.0.63353-0 15774679

[2] Parte AC (2014). LPSN--list of prokaryotic names with standing in nomenclature. Nucleic Acids Res.

[3] Qin S, Zhao GZ, Li J, Zhu WY, Xu LH, Li WJ. *Jiangella alba* sp. nov., an endophytic actinomycete isolated from the stem of *Maytenus austroyunnanensis*. Int J Syst Evol Microbiol. 2009;59:2162–5. 10.1099/ijs.0.009001-0 19605701

[4] Lee SD. *Jiangella alkaliphila* sp. nov., an actinobacterium isolated from a cave. Int J Syst Evol Microbiol. 2008;58:1176–9. 10.1099/ijs.0.65479-0 18450709

[5] Kämpfer P, Schafer J, Lodders N, Martin K. *Jiangella muralis* sp. nov., from an indoor environment. Int J Syst Evol Microbiol. 2011;61:128–31. 10.1099/ijs.0.022277-0 20173006

[6] Suksaard P, Duangmal K, Srivibool R, Xie Q, Hong K, Pathom-Aree W. *Jiangella mangrovi* sp. nov., isolated from mangrove soil. Int J Syst Evol Microbiol. 2015;65:2569–73. 10.1099/ijs.0.000303 25948618

[7] Stackebrandt E, Rainey FA, Ward-Rainey NL. Proposal for a new hierarchic classification system, *Actinobacteria* classis nov. Int J Syst Bacteriol. 1997;47:479–91.

[8] Zhi XY, Li WJ, Stackebrandt E. An update of the structure and 16S rRNA gene sequence-based definition of higher ranks of the class *Actinobacteria*, with the proposal of two new suborders and four new families and emended descriptions of the existing higher taxa. Int J Syst Evol Microbiol. 2009;59:589–608. 10.1099/ijs.0.65780-0 19244447

[9] Tang SK, Zhi XY, Wang Y, Shi R, Lou K, Xu LH, et al. *Haloactinopolyspora alba* gen. nov., sp. nov., a halophilic filamentous actinomycete isolated from a salt lake, with proposal of *Jiangellaceae* fam. nov. and *Jiangellineae* subord. nov. Int J Syst Evol Microbiol. 2011;61:194–200. 10.1099/ijs.0.021725-0 20190023

[10] Goodfellow M. Class I. *Actinobacteria*. In: GM G, DJ B, NR K, JT S, editors. Bergey’s Manual of Systematic Bacteriology, vol. 5. 2nd ed. New York: Springer; 2012. p. 34.

[11] Han L, Gao C, Jiang Y, Guan P, Liu J, Li L, et al. Jiangrines A-F and jiangolide from an actinobacterium,* Jiangella gansuensis*. J Nat Prod. 2014;77:2605–10. 10.1021/np500402a 25412141

[12] Shirling EB, Gottlieb D. Methods for characterization of *Streptomyces* species1. Int J Syst Evol Microbiol. 1966;16:313–40.

[13] Kim OS, Cho YJ, Lee K, Yoon SH, Kim M, Na H (2012). Introducing EzTaxon-e: a prokaryotic 16S rRNA gene sequence database with phylotypes that represent uncultured species. Int J Syst Evol Microbiol.

[14] Li L, Ma J-B, Abdalla Mohamad O, Li S-H, Osman G, Li Y-Q, et al. *Phytoactinopolyspora endophytica* gen. nov., sp. nov., a halotolerant filamentous actinomycete isolated from the roots of *Glycyrrhiza uralensis* F. Int J Syst Evol Microbiol. 2015;65:2671–7. 10.1099/ijs.0.000322 25964514

[15] Göker M, Klenk HP (2013). Phylogeny-driven target selection for large-scale genome-sequencing (and other) projects. Stand Genomic Sci.

[16] Kyrpides NC, Hugenholtz P, Eisen JA, Woyke T, Göker M, Parker CT (2014). Genomic Encyclopedia of *Bacteria* and *Archaea*: sequencing a myriad of type strains. PLoS Biol.

[17] Klenk HP, Göker M. En route to a genome-based classification of *Archaea* and *Bacteria*? Syst Appl Microbiol. 2010;33:175–82. 10.1016/j.syapm.2010.03.003 20409658

[18] Wu D, Hugenholtz P, Mavromatis K, Pukall R, Dalin E, Ivanova NN (2009). A phylogeny-driven genomic encyclopaedia of Bacteria and Archaea. Nature.

[19] Liolios K, Mavromatis K, Tavernarakis N, Kyrpides NC (2008). The Genomes On Line Database (GOLD) in 2007: status of genomic and metagenomic projects and their associated metadata. Nucleic Acids Res.

[20] Mavromatis K, Land ML, Brettin TS, Quest DJ, Copeland A, Clum A, Goodwin L, Woyke T, Lapidus A, Klenk HP, Cottingham RW, Kyrpides NC (2012). The fast changing landscape of sequencing technologies and their impact on microbial genome assemblies and annotation. PLoS ONE.

[21] Field D, Garrity G, Gray T, Morrison N, Selengut J, Sterk P (2008). The minimum information about a genome sequence (MIGS) specification. Nat Biotechnol.

[22] Hyatt D, Chen GL, Locascio PF, Land ML, Larimer FW, Hauser LJ (2010). Prodigal: prokaryotic gene recognition and translation initiation site identification. BMC Bioinformatics.

[23] Mavromatis K, Ivanova NN, Chen IM, Szeto E, Markowitz VM, Kyrpides NC (2009). The DOE-JGI Standard operating procedure for the annotations of microbial genomes. Stand Genomic Sci.

[24] Chen IM, Markowitz VM, Chu K, Anderson I, Mavromatis K, Kyrpides NC, Ivanova NN (2013). Improving microbial genome annotations in an integrated database context. PLoS ONE.

[25] Pati A, Ivanova N, Mikhailova N, Ovchinikova G, Hooper SD, Lykidis A, Kyrpides NC (2010). GenePRIMP: A Gene Prediction Improvement Pipeline for microbial genomes. Nat Methods.

[26] Finn RD, Clements J, Eddy SR (2011). HMMER web server: interactive sequence similarity searching. Nucleic Acids Res.

[27] Lowe TM, Eddy SR (1997). tRNAscan-SE: a program for improved detection of transfer RNA genes in genomic sequence. Nucleic Acids Res.

[28] Nawrocki EP, Kolbe DL, Eddy SR (2009). Infernal 1.0: inference of RNA alignments. Bioinformatics.

[29] Markowitz VM, Mavromatis K, Ivanova NN, Chen IM, Chu K, Kyrpides NC (2009). IMG ER: a system for microbial genome annotation expert review and curation. Bioinformatics.

[30] Bland C, Ramsey TL, Sabree F, Lowe M, Brown K, Kyrpides NC (2007). CRISPR recognition tool (CRT): a tool for automatic detection of clustered regularly interspaced palindromic repeats. BMC Bioinformatics.

[31] Petersen J (2011). Phylogeny and compatibility: plasmid classification in the genomics era. Arch Microbiol.

[32] Hildebrand F, Meyer A, Eyre-Walker A (2010). Evidence of selection upon genomic GC-content in bacteria. PLoS Genet.

[33] Wu H, Zhang Z, Hu S, Yu J (2012). On the molecular mechanism of GC content variation among eubacterial genomes. Biol Direct.

[34] Ranea JA, Buchan DW, Thornton JM, Orengo CA (2004). Evolution of protein superfamilies and bacterial genome size. J Mol Biol.

[35] Burke GR, Moran NA (2011). Massive genomic decay in Serratia symbiotica, a recently evolved symbiont of aphids. Genome Biol Evol.

[36] Hadjithomas M, Chen IM, Chu K, Ratner A, Palaniappan K, Szeto E (2015). IMG-ABC: A Knowledge Base To Fuel Discovery of Biosynthetic Gene Clusters and Novel Secondary Metabolites. MBio.

[37] Medema MH, Blin K, Cimermancic P, de Jager V, Zakrzewski P, Fischbach MA (2011). antiSMASH: rapid identification, annotation and analysis of secondary metabolite biosynthesis gene clusters in bacterial and fungal genome sequences. Nucleic Acids Res.

[38] Blin K, Medema MH, Kazempour D, Fischbach MA, Breitling R, Takano E (2013). antiSMASH 2.0--a versatile platform for genome mining of secondary metabolite producers. Nucleic Acids Res.

[39] Tamura K, Peterson D, Peterson N, Stecher G, Nei M, Kumar S (2011). MEGA5: molecular evolutionary genetics analysis using maximum likelihood, evolutionary distance, and maximum parsimony methods. Mol Biol Evol.

[40] Euzeby JP (1997). List of Bacterial Names with Standing in Nomenclature: a folder available on the Internet. Int J Syst Bacteriol.

[41] Garrity G. NamesforLife. BrowserTool takes expertise out of the database and puts it right in the browser. Microbiol Today. 2010;37:9.

[42] Woese CRKO, Wheelis ML (1990). Towards a natural system of organisms: Proposal for the domains Archaea, Bacteria, and Eucarya. Proc Natl Acad Sci U S A.

[43] Garrity G, Holt J, Garrity G, Boone D, Castenholz R (2001). The Road Map to the Manual. Bergey’s Manual of Systematic Bacteriology.

[44] Tang SK, Zhi XY, Li WJ. Order VIII. *Jiangellales* ord. nov. In: Goodfellow M, Kämpfer P, Busse HJ, Trujillo ME, Suzuki K, Ludwig W, Whitman WB, editors. Bergey’s Manual of Systematic Bacteriology, vol. 5. 2nd ed. New York: Springer; 2012. p. 555.

[45] Ashburner MBC, Blake JA, Botstein D, Butler H, Cherry JM, Davis AP, Dolinski K, Dwight SS, Eppig JT (2000). Gene ontology: tool for the unification of biology: the gene ontology consortium. Nat Genet.

[46] Grant JR, Stothard P (2008). The CGView Server: a comparative genomics tool for circular genomes. Nucleic Acids Res.

