# Supplementary figures and images for: Complete genome sequence of Jiangella gansuensis strain YIM 002T (DSM 44835T), the type species of the genus Jiangella and source of new antibiotic compounds
Source: Stand Genomic Sci. 2017 Feb 3;12:21. doi: 10.1186/s40793-017-0226-6 (PMC5292007; doi:10.1186/s40793-017-0226-6)

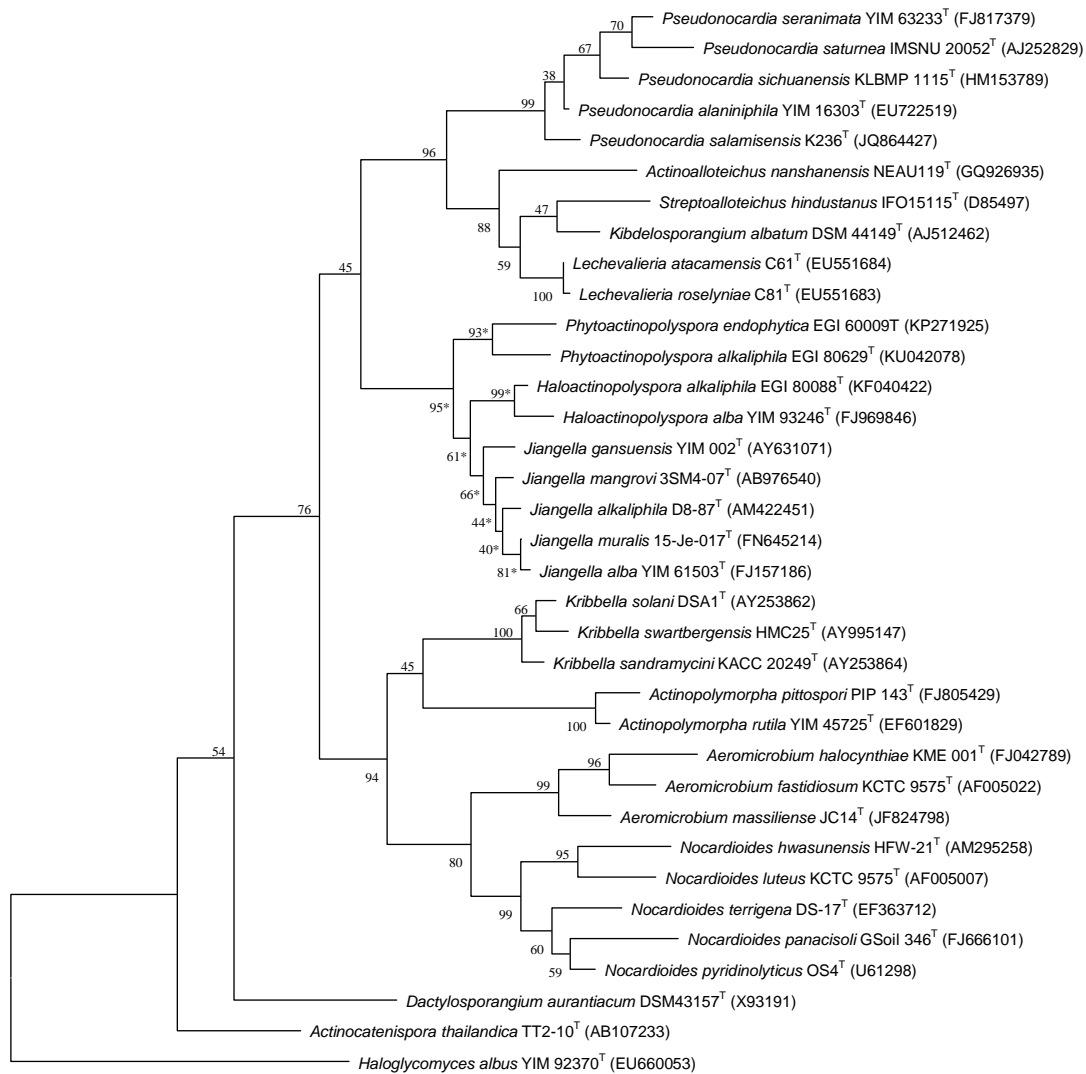

0.02

Supplement: Additional file 1: Figure S1. — Phylogenetic tree showing the relationship of J. gansuensis YIM 002T with some other actinobacteria based on 16S rRNA sequences. The maximum-likelihood tree was built using MEGA 5 [39]. Bootstrap values (percentages of 1000 replicates) are shown at branch points. Haloglycomyces albus was used as outgroup. (PDF 92 kb) [file 40793_2017_226_MOESM1_ESM.pdf]
