# Supplementary material for: Complete genome sequence of Jiangella gansuensis strain YIM 002T (DSM 44835T), the type species of the genus Jiangella and source of new antibiotic compounds
Source: Stand Genomic Sci. 2017 Feb 3;12:21. doi: 10.1186/s40793-017-0226-6 (PMC5292007; doi:10.1186/s40793-017-0226-6)
Supplement: Additional file 2: Table S1. — Number of gene clusters associated with antiSMASH. (DOCX 73 kb) [file 40793_2017_226_MOESM2_ESM.docx]

**Table S1** Number of gene clusters associated with antiSMASH

| **Type^a^** | **Number^b^** | **Most similar known cluster^c^** |
| --- | --- | --- |
| Cf_saccharide | 16 | A201A biosynthetic gene cluster |
|  |  | Hedamycin biosynthetic gene cluster |
|  |  | Pristinamycin biosynthetic gene cluster |
|  |  | Streptomycin biosynthetic gene cluster |
|  |  | Phosphonoglycans biosynthetic gene cluster |
| Cf_fatty_acid | 3 | R1128 biosynthetic gene cluster |
|  |  | Asukamycin biosynthetic gene cluster |
| Siderophore | 1 | - |
| Lantipeptide | 1 | Catenulipeptin biosynthetic gene cluster |
| T3pks | 1 | Alkylresorcinol biosynthetic gene cluster |
| Lassopeptide | 1 | 5’-hydroxystreptomycin biosynthetic gene cluster |
| Cf_saccharide-lantipeptide | 1 | - |
| Cf_putative | 36 | Lividomycin biosynthetic gene cluster |
|  |  | Enduracidin biosynthetic gene cluster |
|  |  | Thiolutin biosynthetic gene cluster |
|  |  | Ochronotic pigment biosynthetic gene cluster |
|  |  | Quartromicin biosynthetic gene cluster |
|  |  | Streptomycin biosynthetic gene cluster |
|  |  | Meilingmycin biosynthetic gene cluster |
|  |  | Necocarzinostatin biosynthetic gene cluster |
|  |  | K-252a biosynthetic gene cluster |
|  |  | Desotamide biosynthetic gene cluster |

a) Type is consistent with the library of models specific for describe the gene clusters in antiSMASH

b) The total number of annotated gene clusters.

c) Only list the gene clusters identified to the most similar known cluster.
